# National Disaster Management System: COVID-19 Case in Korea

**DOI:** 10.3390/ijerph17186691

**Published:** 2020-09-14

**Authors:** Junic Kim, Kelly Ashihara

**Affiliations:** School of Business, Konkuk University, 120 Neungdong-ro, Seoul 05029, Korea; kashihara@konkuk.ac.kr

**Keywords:** national control tower, disaster management, COVID-19, pandemic, disease

## Abstract

The COVID-19 pandemic poses unprecedented challenges for governments and societies around the world and represents a global crisis of hitherto unexperienced proportions. Our research seeks to analyse disaster management systems from a national perspective by examining the Korean management of the COVID-19 crisis according to a four-phase epidemiological disaster management system. Utilising a meta-study, official documents, reports and interviews, we explore the role of the control tower mechanism related to the life-cycle of disaster management, and Korea’s sustainable containment strategy. This study begins with a discussion of the crisis and disaster management literature and provides specific information related to the Korean government’s response to COVID-19. It continues by detailing specific strategies such as wide-spread testing, tracking, treatment and quarantine that have enabled Korea to prevent wide-spread community transmission. The study concludes emphasising the relevance of systematic national disaster management, providing insight into methods for containment in Korea – a system commended by the WHO. Implications include the extension and the efficient application of disaster management theory by empirical application and integration of concepts.

## 1. Introduction

A nation’s disaster management system encompasses a wide range of functions, depending on the level of significance assigned to a particular crisis. In the past, these functions mainly included the management of natural disasters like floods and typhoons; however, since the early 1990s, man-made disasters such as building collapses, gas leaks, and ship sinking accidents have also come under the auspices of disaster management. Therefore, man-made disaster management systems have also received attention in this new phase. Today, new types of disaster, entirely different from conventional disasters, are threatening our society: information and communication disruptions, paralysis of transportation and transportation systems, terrorism, and epidemics. In particular, as global health disasters such as COVID-19, SARS, and MERS threaten the international community, the necessity to establish a disaster management system from a national perspective has become paramount. In other words, the paradigm of national disaster management is changing into various new forms. In addition to the massive cost in terms of lives lost and economic upheaval, there has been a complete and utter interruption of the regular pattern of life in terms of education, work, and public transport—not to mention, an unprecedented impact on healthcare systems. When faced with such a crisis of unknown magnitude and epidemiology, citizens look to their governments for leadership, guidance and information. Therefore, the government should move away from passive activities and deal with the situation more actively and systematically at the national level.

The level of pervasive contagion and impact on society of the global COVID-19 pandemic is markedly different than that of SARS and MERS. which recently made the world a pandemic. Since December 2019—when the first COVID-19 cases were detected in Wuhan, China, unbeknownst to the rest of the world—mass contagion was underway. On 31 January 2020, the World Health Organisation (WHO) declared an international public health emergency, and on February 28 the WHO raised the global risk level of COVID-19 to “very high.” On March 11, the WHO declared COVID-19 a global pandemic. As of June 2020, six months after the initial outbreak in Wuhan, the world has experienced mass infection, with nearly sixteen million positive cases and 650,000 deaths spread across all the continents. The COVID-19 pandemic is posing an unprecedented leadership challenge for governments worldwide—the kind of challenge that has been historically restricted to situations of war or serious natural disasters. In addition to the massive cost in terms of lives lost and economic upheaval, there has been a complete interruption of the regular pattern of life in terms of education, work, and public transport—not to mention, an unprecedented impact on healthcare systems. When faced with such a crisis of unknown magnitude and epidemiology, citizens look to their governments for leadership, guidance, and information While the different political ideologies of socialism, communism, and capitalism in the post-World War II era have led to divergent views on the roles of government, in times of crisis it is clear that the government has the role and the responsibility to protect citizens from harm. Clearly, the ability to respond quickly, sensibly, and responsibly to a wide range of emergencies is a “must-have” for political leaders. This study adopts traditional crisis management theory to include four distinct phases. In particular, the study analyses the actions and strategic responses of Korea’s national disaster management system in each phase —evaluated as an excellent case of disaster management by the WHO.

The duration of the study is as follows: from 20 January, when the first COVID-19 case occurred in Korea, to February 18, when the number of infected increased exponentially because of super-spreaders; and from March 22, when strict social distancing began, to 5 May, when strict social distancing requirements were lifted and routine distancing was restored. The first phase lasts from 19 January to 18 February; the second phase from 19 February to 21 March; the third phase from 22 March to 5 May; and the fourth phase, which started on 6 May, is ongoing. The world suffers several major and minor disasters every year. Therefore, it is imperative that governments—which are obliged to protect the lives and properties of their people—put in place preventive measures in anticipation of expected or impending disasters, and establish systems of prompt emergency recovery and restoration in the event of a disaster. The purpose of this study is to explore how to establish an effective governmental disaster management system for each stage. Therefore, we examine the Korean disaster management system and analyse the features of its system.

## 2. Theoretical Background

Disaster management refers to the development and implementation of policies on disaster prevention, planning, response, and recovery to minimise damage caused by disasters [1]. National disasters are chaotic situations that threaten the existing social order. As a disaster unfolds and the situation is exacerbated, known reality undergoes a drastic change and the ability of the public to comprehend and attribute meaning to what is happening is seriously challenged. If the crisis persists, it poses a complex threat not only to human lives, but to the systemic cooperation that links authorities and institutions. Crisis management is potentially dangerous for the control, reputation, and public legitimacy of leadership. For the purposes of this paper, we define disaster as “a serious threat to the basic structures or the fundamental values and norms of a social system, which—under time pressure and highly uncertain circumstances—necessitates making critical decisions.” [2]. Gilbert [3] stresses that a disaster is an attack from the outside that interferes with the social system, with three models believed to exist. The “repression of social vulnerability” model showed that disasters were caused by vulnerabilities inherent in the internal processes of a society. In addition, the expression ”entrance into a state of uncertainty“ means that a disaster is a result of the failure to identify real or hypothetical risks and occurrences, when the conventional framework for understanding reality stops functioning. Posner [4] (2004) suggests that, for effective disaster management, a crisis should be classified into one of four types. In other words, national disasters have unique characteristics, unlike those of organisations or systems. Therefore, the response to national disasters needs to be designed with the disaster’s unique characteristics in mind. As Turner [5] notes, disaster management is the result of the time-specific exposure to risk factors that have accumulated over a long period since the onset of visibility. According to the man-made disaster model, technical, social, institutional, and administrative devices cause disasters [5,6].

In other words, the environment surrounding a disaster management administrative system is characterised by interaction, uncertainty, and complexity, unlike a typical administrative environment [7]. This “interaction” refers to that between the causes of the disaster and the factors that spread the damage. Alternatively, disasters generally do not result from a single cause. Even if there is a specific, definitive cause, this has a reciprocal synergistic effect on the occurrence of other factors. Eventually, this interaction will determine the overall intensity and scope of the damage. Thus, disasters in modern societies are increasing the density and complexity of their interactions, with one type of disaster causing another, and vice versa [8]. Another characteristic of disaster is “uncertainty,” which means that the nature of the disaster may change, and disaster management organisation and existing disaster response must, therefore, change accordingly [9].

Alternatively, factors may be highly uncertain because the nature of the disaster can change. It is also unclear how much damage an accident will cause. Disasters have uncertainty of “occurrence” and “progress”, because “occurrence” of disasters usually exists outside the forecast range. Even if disaster factors are minimised, disaster occurrence is inevitable. These theoretical frameworks for disaster management can be largely divided into two categories: the disaster management strategy and the disaster management process. First, the disaster management strategy is divided into prevention and restoration strategies, focusing on theoretical discussions on risks and uncertainties [10]. The Disaster Management process is usually divided into four stages, mitigation, preparedness, response, and recovery, based on the time of the disaster [9,11,12]. Mitigation refers to pre-risk preparedness [13], activities which eliminate or suppress disaster triggering factors before a catastrophe occurs [11,14]. Preparedness refers to the ability to develop response capabilities in advance for response activities in the event of a crisis [11,12]. These two stages require close interconnection [9]. Response is an activity that applies the various duties and functions of disaster management agencies after the disaster has occurred [9,11], and recovery refers to the long-term course of activity for healing in the designated area.

It is imperative to realise that damage may be minimised by pre-emptive disaster preparation, centralisation of decision-making and efforts to mitigate damage. The scale of disaster impact and damage is also difficult to confirm, as disasters are by nature unpredictable, even after their occurrence, especially as one disaster is likely to lead to a chain of subsequent new disasters, as disasters are known to interact [15,16,17]. Among the characteristics of a disaster, “complexity” refers to the restructure and relationship among the agencies involved after the occurrence of a disaster. A disaster is characterised by complicated processes, factors, causes, and restorative actions. This complexity arises as factors related to the progression of a disaster, directly and indirectly, affect other factors. This is also linked to the aforementioned “interaction.“ Therefore, it is critical to identify the elements of disaster management systems that should be equipped so as to enhance the effectiveness of response management in environments that are under strain and affected by the factors of interaction, uncertainty, and complexity [18]. Contemporarily, the disaster management literature has also evolved, with companies increasingly becoming transnational and technology and platforms [19,20] creating new types of interdependency.

As a result of this increasingly complex nature of disaster management, there is a movement to manage disasters from a national perspective systematically. At the nation-state level, disaster management encompasses numerous functions depending on the level of significance assigned to a particular crisis. While there is some precedent of infectious diseases such as SARS, MERS, and Ebola, given the mass contagion underway in the United States and Europe questions arise as to the best practices for COVID-19 containment and the key success factors for crisis management. National disaster management, in particular, is a complicated process requiring analysis and management within the lifecycle of disasters. Within existing studies, there are a few disaster cycle studies, which are often divided into mitigation-planning-response-recovery before and after a disaster occurs. However, there is little research on how to deal with disasters periodically after their occurrence, as many national disasters seem to be finite in location time and circumstance. In addition, there is a paucity of research on disaster management systems from a national perspective. Given the duration and persistence of COVID-19, there is still urgency and relevancy in understanding best practices. In this study, we present the case study of Korea’s national disaster management mechanism in response to COVID-19, which was commended by the WHO.

## 3. Research Design

### 3.1. Research Model

For the purposes of this paper, we define crisis as “a serious threat to the basic structures or the fundamental values and norms of a social system, which—under time pressure and highly uncertain circumstances—necessitates making critical decisions.” [21]. In search of a relevant model to analyse a wide range of disaster management activities, we first evaluated relevant crisis management models including the onion model for strategic crisis of Pauchant and Mitroff [22] and the crisis management model of Mitroff [23]. Pauchant and Mitroff [22]’s onion model categorises organisations that are vulnerable to a crisis and those that are prepared for the crisis, and the model of Mitroff [23] divides the disaster situation into pre-crisis and post-crisis. After reviewing these models, therefore, we determined that they were not suitable for the time development pattern of a crisis situation. Similarly, we considered Coppola’s [24] approach to the crisis management team and Carter’s [25] international disaster management model; however, none conformed to the event trends related to the need for central control and government. Fink’s [26] four-stage model examines crisis as an extended event with sufficient warning signs that precede the event: the prodromal stage, the acute stage, the chronic stage, and the resolution stage. However, there was a lack of explanation of the important characteristics and elements of each stage. We also considered the three-step model of Coombs [27] but ruled it out as specifics related to issue management were neglected. Based upon a meta-analysis, we concluded that the crisis management process model was most suitable as it includes the analysis of the development process, i.e., the issue’s life-cycle. As disaster management has generally focused on the metaphor of a crisis’s “life-cycle,” we opted to revise the existing life-cycle models [26,27,28] and developed these models to deal with a disaster after its occurrence.

**Figure 1:**
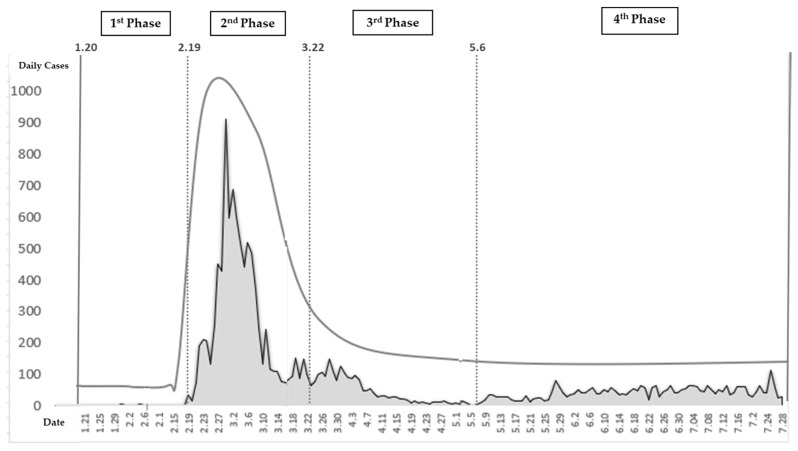
Disaster management process for COVID-19 in Korea. Source: Authors’ Elaboration.

This study creates a four-phase epidemiological disaster management system (See Figure 1). In other words, depending on the life-cycle of disasters, this study classified the four phases of the disaster management system as proactive, reactive, emergency response, and recovery, which then is recycled by integration of best practice through post-mortem analysis [29]. The proactive phase conducts programs that reduce the likelihood of a crisis by considering the risks and the possible responses upon early discovery. It is necessary to collect, evaluate, and develop such strategies. The reactive phase includes preparation of an operational plan to handle an emergency or to maintain the ability to respond to a crisis whose likelihood is high despite mitigation efforts. In particular, this is the phase when damage from a disaster increases explosively. The response phase is the most crucial as this is when steps are taken in response to the crisis being at its peak. The recovery phase refers to the period when the damage is first reduced and there is recovery. The subject of this study, COVID-19, was considered the most suitable risk management process model because it has a time-ordered nature (See Figure 1 and Supplementary Table S1 and Table S2).

### 3.2. Data Collection

By 29 June 2020, there were over 10,000,000 confirmed positive cases of COVID-19 worldwide; in South Korea, there were 14,251 confirmed cases with 300 deaths (See Table 1). In the meantime, confirmed cases had been reported in all of Korea’s 17 metropolitan cities and provinces. In particular, 59% of total cases occurred in Daegu and North Gyeongsang Province, where the infection explosion was caused by a religious organisation called Sincheonji. These data were collected from three main sources. The first source is Korean government documents. This study analysed and utilised official documents, reports, and news items from the Korea Centers for Disease Control and Prevention (KCDC); the Ministry of Health and Welfare; the National Assembly of the Republic of Korea; the Ministry of Culture, Sports, and Tourism; the National Research Institute of Public Health and Environment; and the Commissioner of Statistics, Korea. The second source are web portals. We collected data by inserting specific keywords into Naver, the largest web portal in Korea, and Kakao, a mobile platform. Keywords were collected by inputting Korean and English words in parallel, such as COVID-19, Corona, coronavirus, and Wuhan virus.

Data were collected for four specific periods: the first period starts from 20 January 2020, when the first COVID-19 positive patient was reported in Korea; the second period starts from 19 February 2020, when the number of positive cases increased exponentially because of super-spreaders; the third period starts from 22 March 2020, when strict social distancing began; and the last period starts from 6 May 2020, when strict social distancing requirements were lifted and routine distancing was restored. This study sought to use previous research on disaster response to list the factors affecting such response and to present theoretical frameworks and related information. Thus, factors affecting disaster response were reviewed in existing studies during the theory-building process and organised at the system theory level. A meta-study was conducted to explore policies, articles, and interviews with the Korean government. A protocol was built through data collection and analysis, which tried to ensure the reliability and validity of the research. This study was analysed systematically from the data collection stage to the final analysis stage. We also studied specifics related to the Korea’s control tower mechanism, which we believe is directly related to Korea’s sustainable containment strategy (See supplementary materials).

To verify secondary data, this study also conducted twelve focus groups based upon three sections of a business communications, leadership and negotiations course at a well-known Korean University and its discussion on COVID-19. Based upon the outcomes, we selected and interviewed six individuals based on their experiences in testing, quarantine, or careers. All interviews were recorded and conducted via Zoom, with detailed notes and approval from interviewees relating to discussed content. Students asked the same five questions related to perspective, testing, quarantine, compliance, and government roles in handling COVID-19; professional questions were tailored to their respective specialty. Interviews were primarily for the purposes of validating policies and understanding specifics related to control tower implementation, as many has been officially quarantined and had had first-hand experience of individuals who had COVID-19. This process validated our insights and provided subtle nuances relating to theory, Korean government official communication and actual implementation of the policy.

## 4. Findings

### 4.1. 1*st* Phase (20 January–18 February)

When the first few confirmed cases of COVID-19 were reported in South Korea, the government acted swiftly, action that has been correlated with reaction to the COVID crisis seen in many countries. On 31 December 2019, 27 patients with pneumonia were reported in Wuhan, Hubei Province, China—all of them had visited a wet market in Wuhan. The KCDC, which determined that the new infectious disease had originated overseas, formed the “Task Force for COVID-19” in Wuhan, China on 3 January 2020, and designated the crisis as “Attention (Blue).” Accordingly, the Commission continued to identify overseas trends and began monitoring and responding to suspected cases. In the event of a suspected case, a basic epidemiological survey was conducted by a public health center, and the central epidemiological investigator decided whether to conduct an examination after the patient had been admitted to a state-designated sound pressure bed, or whether to implement management measures such as active monitoring. In addition, from January 8th onward, Wuhan was designated as a contaminated area, and a health status questionnaire was created for those who arrived in Wuhan. If they had a body temperature of 37.5 degrees Celsius or more, or if they had respiratory symptoms such as coughing, they were quarantined.

**Figure 2:**
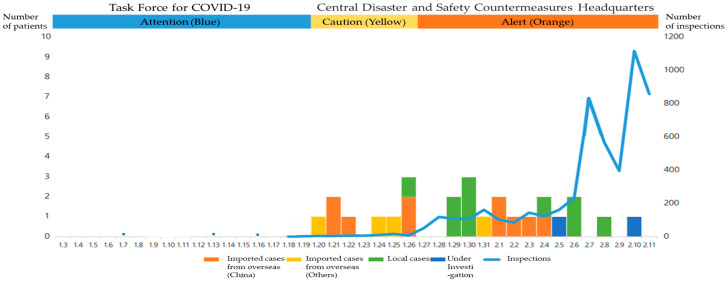
The epidemic curve for COVID-19 in Korea (by date of onset as of February 11, 2020).

**Figure 3:**
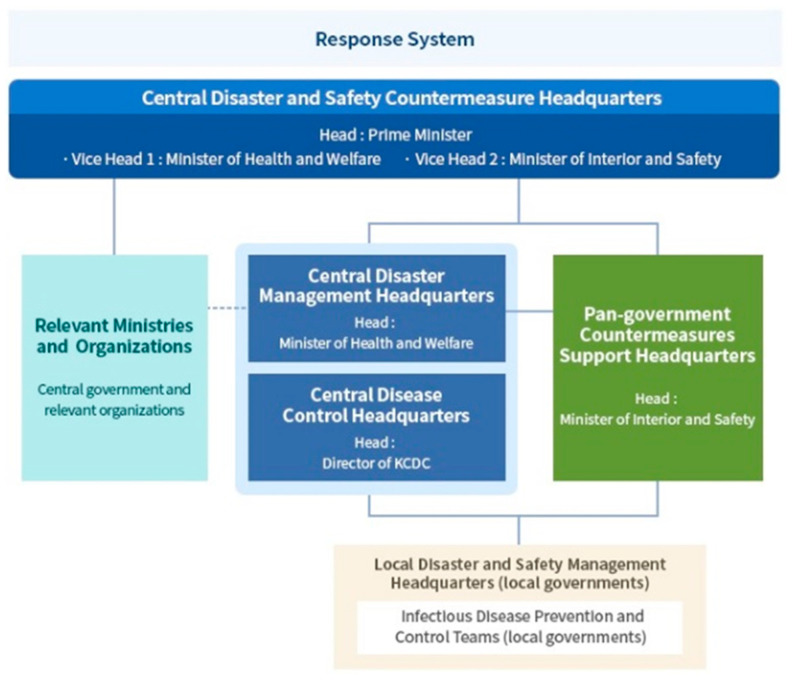
Korean government’s response system. (Central Disease Control Headquarters; National Control Tower).

Phase 1 goes from the first confirmed COVID-19 case reported in Korea on 20 January 2020 to the super spreader incident, when the number of infections exponentially increased. Upon identification of the first case in Korea on January 20th, having planned for a potential crisis, the KCDC immediately upgraded the designation of the crisis from “Attention (Blue)” to “Caution (Yellow)” and began intensive management (See Figure 2). It began intensive contact tracking and developed a system of thorough examination and treatment—even the asymptomatic were quarantined to check for infection. The first patient was picked up from an airport in Korea and immediately isolated; the second patient was classified as an active surveillance subject during the quarantine process and was monitored by health authorities. The KCDC analysed the epidemiological and initial clinical characteristics of patients who were identified using COVID-19 reporting and surveillance data and a field response report from the rapid response team (See Figure 3). Additionally, merely a week after the first confirmed cases were reported, the Korean government met with representatives of medical device manufacturers, urging the development of diagnostic kits and promising approval for emergency use. Consequently, within two weeks, when the number of confirmed patients in Korea was less than 30, thousands of diagnostic kits were ready.

Moreover, the Korean government quickly built a national control tower, the “Central Disaster Safety and Countermeasure Headquarters,” headed by the prime minister. Given the distinct nature and expertise involved in responding to an infectious disease, the “Central Disease Control Headquarters” is led by the director of the KCDC. As for those entering Korea, the protocol to deal with symptomatic as well as asymptomatic passengers was well in place. The Korean government had detected confirmed cases early and had blocked the spread of the virus through cooperation with medical institutions. Since the outbreak, the director of the KCDC, an expert on infectious diseases, has largely been in charge of the situation, overseeing the implementation of the ”COVID-19 quarantine model” that has significantly contributed to Korea’s low fatality rate, which is one of the lowest worldwide. Inspections and diagnoses, tracking skills citizenship training, wearing masks and public cooperation were cited as reasons for the success of the COVID-19 quarantine model.

### 4.2. 2*nd* Phase (19 February–21 March)

In Korea, community transmission started when COVID-19 confirmed cases rose from 31 cases on 18 February to 46 cases on 19 February, and to 104 cases confirmed cases by 20 February. In the context of community transmission, through contact tracking and epidemiological investigation, the Korean authorities identified “super spreader” incidents. Initially, one well known case was a Shincheonji church member who was a super spreader and infected several people at the Shincheonji Church in Daegu and Gyeongbuk provinces and at least 37 others, leading to an increase from 130 cases on 25 February to 909 confirmed cases four days later on 29 February. Upon identification of the mass contagion in Daegu and Gyoengbuk provinces, the Korean national control tower raised the crisis level from ‘Alert (Orange)’ to ‘Serious (Red)’ on 24 February. The Korean government conducted mass testing early, frequently and safely to keep community contagion from worsening. Kang Kyung-wha, the Minister of Foreign Affairs, said in an interview with the BBC that the importance of testing was the key to enabling Korea to have a low fatality rate because it enabled early detection of further infection and the rapid treatment of infected people. Korea has tested approximately 2.1 million or 4.05% of the population as of 10 September 2020, which is one of the highest ratios in the world.

**Figure 4:**
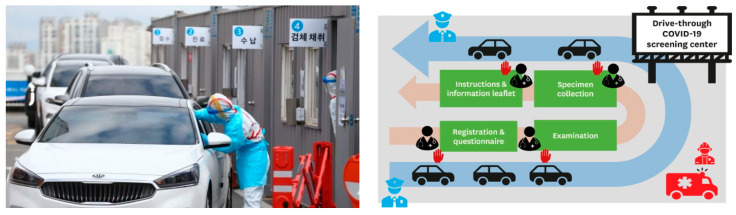
Drive-through centres for COVID-19.

**Figure 5:**
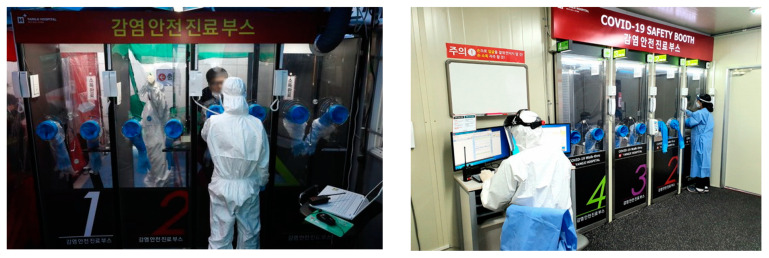
COVID-19 Safety Booth (Walk-through centres) for COVID-19.

Under the direction of the KCDC, the Korean Government swiftly established over 600 inspection centers to prevent hospitalisation and keep medical personnel safe by minimising contact between staff and patients. Test results were available within a few hours from 50 drive-through centres with tests conducted within ten minutes while sitting in a car. Similarly, walk-in centers resembled transparent telephone booths. This system earned much global praise. Korea also innovatively developed ‘walk-through’ testing booths in order to overcome the drive-through method problem, which requires a large space in order to test people in their vehicles. Walk-through testing is based on a one-person screening booth that separates doctors and patients, reducing the risks of infection and allowing seniors and patients without cars to walk through the box and be tested. Figure 4 and Figure 5 indicate walk-through and drive-through testing facilities in Korea.

**Figure 6:**
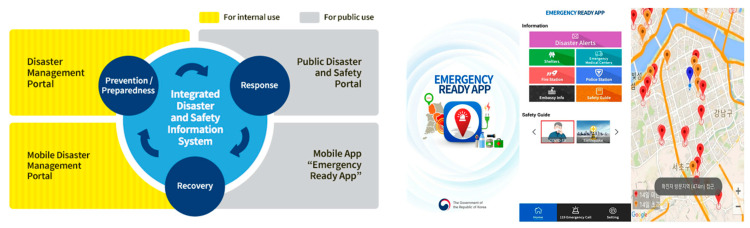
Korean integrated disaster safety and information system, and emergency alerts.

The Korean government opted for information disclosure. Dissemination of information included sharing information regarding the mobilisation of the medical community and disclosure of raw data for analysis by experts, scholars and doctors. In terms of COVID-19, this involved mathematical modeling and the assessment of infection, susceptibility, treatment, mortality, and the willingness to be accountable for results. As part of the efforts to establish transparent communication, the Korean government established the COVID-19 website to communicate with the public and press on February 5th and, capitalising on one of the highest per capita smartphone penetration rates of 95%, the Korean Government activated the emergency alert message system which broadcasts an average of 20 alerts a day including social distancing rules to follow and updates on newly diagnosed patients in the area. While the daily broadcast of so many alerts disturbed citizens’ lives, it served the purpose of putting the COVID-19 crisis at the forefront. These emergency alert text messages announced the locations which confirmed COVID-19 patients had visited nearby. Figure 6 indicates the integrated disaster safety information system and emergency alerts.

### 4.3. 3*rd* Phase (22 March–5 May)

**Figure 7:**
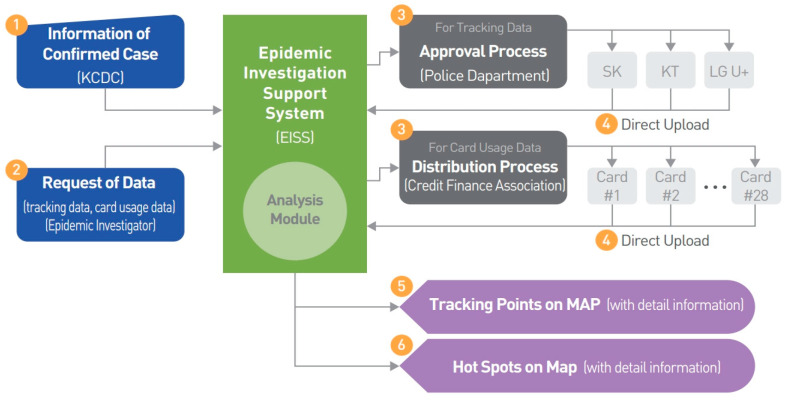
Korea’s epidemiological investigation support system [30].

As the number of infected people continued to increase, the Korean government implemented the “social distancing” rule from March 22nd. It was recommended to limit the operation of some high-risk facilities and industries by staying at home as much as possible and to minimise contact amongst colleagues. The government also urged the public to observe personal hygiene practices such as regular washing of hands and coughing with the mouth covered and to refrain from attending activities such as religious events or visiting indoor sports facilities, which could lead to close contact in tight spaces. In addition, contact tracking and epidemiological investigations, containment and monitoring were continuously conducted. To this end, the government has established a system for supporting epidemiological investigations of COVID-19 by using smart city technology (See Figure 7). This system collects big data such as the mobile phone location information and the credit card usage history of an individual and derives their movements within ten minutes. Immediately after the contagion began, the Ministry of Land, Infrastructure and Transport began working on the development of this system and developed and transferred the system to the KCDC within a month. The Korean government has already established a “smart city data hub platform” and can analyse various kinds of big data in real-time such as data concerning traffic, energy, environment, and safety in cities. Previously, an epidemiological investigator had to make a phone call to the relevant institution to obtain the desired information and data in order to analyse movement. Through this new system, real-time data was exchanged among 28 related institutions to ascertain the movement within ten minutes. Confirmed COVID-19 positive cases are tracked by trained medical staff to locate and examine their potential contacts and isolate them, if necessary—this is called contact tracking investigation. Through this system, the results of interviews with confirmed patients can be complemented, and real-time analysis of big data is possible so that the movement of confirmed cases and their location over time can be automatically identified. In this way, a large-scale outbreak area—or hot spot—is analysed and the source of infection is identified. Additionally, various other statistical analyses are also possible under this system.

In addition, a system was designed to issue notifications detailing the movement of every newly confirmed case in an area. The details include the path traversed by the confirmed patient, the vehicle used, and public transport boarding history, as well as whether the person was wearing a mask. A system was, therefore, established to encourage those whose movements overlapped with the confirmed person’s movements to inform an inspection center. Although issues regarding invasion of privacy were raised, the system was nevertheless implemented. Precedent for this came from the MERS crisis. In the case of MERS, the Ministry of Health considered personal privacy and protected the identity of individuals from healthcare facilities, leading to the release of patients who did not even know they were infected and permission for them to freely move throughout the city. After this crisis, there was a public outcry and, as such, Korea passed a law enabling contract tracing and tracking in the case of epidemics. This highlights the role of mitigation or preparation prior to the first stage of disaster management.

**Figure 8:**
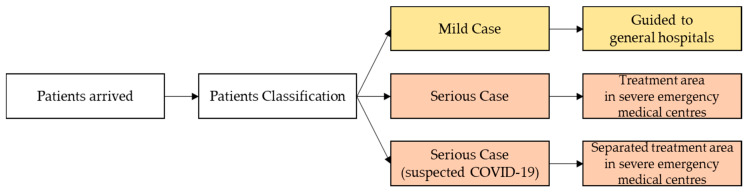
Severe emergency medical centre system.

Furthermore, patients instructed to remain in self-quarantine are required to download an app that alerts the authorities if they violate self-quarantine guidelines, making them liable to pay a fine of approximately $2500. Moreover, Korea was detecting and treating infections early and distinguishing mild patients to send them to special centers, leaving hospitals free for serious patients. Very often, serious patients miss receiving treatment during the critical period—also called the golden time within which treatment can save one’s life—because of inadmissibility to an emergency room (See Figure 8). To ensure that this is not the case with COVID-19 patients, the government set up the “Severe Emergency Medical Centre.” The mortality rate has, therefore, rapidly lowered by designating and operating two or more of these (required) by city or province, and one or more (recommended) by treatment area, 70 in total. Consequently, Korea’s mortality rate today is just over 1.5%—among the lowest in the world. The Regional Emergency Medical Center is intended to treat severe emergency patients professionally and systematically 24 h a day. At the national level, the government divided provinces, cities, and counties into 41 regions and designated one hospital that could play a pivotal role as an emergency medical care and a disaster center in the designated area.

### 4.4. 4*th* Phase (6 May–Current)

**Figure 9:**
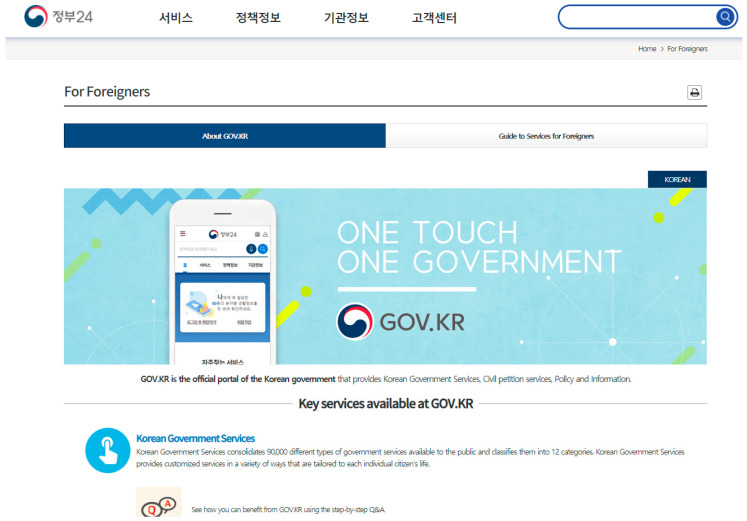
Government 24 System.

As the number of COVID-19 positive cases plummeted, the Korean government quickly began activities to restore the economy and stabilise society. Accordingly, strict social distancing requirements were lifted and routine distancing was restored from May 6. Therefore, in principle, meetings, outings, and events were allowed, and public facilities, hitherto suspended, began to operate step-by-step, first by preparing quarantine guidelines. In addition, dense indoor facilities like national parks, sports facilities, national and public theatres, performance halls, and welfare centers were also opened. Schools and daycare centers were opened in stages. The COVID-19 spread had caused an economic crisis and the domestic economy was jolted by job losses and economic contraction. Consequently, the government initiated several support projects to revive and stabilise the economy. To that end, customised service supports were established through “Government 24”—a public platform (Figure 9). The projects are organised through various government departments and local governments, including the Ministry of Employment and Labor, the Korea Workers’ Compensation & Welfare Service, the National Pension Service, the Ministry of Economy and Finance, and the Small Enterprise and Market Service. Approximately 440 support programmes are organised for individuals and households, and 170 support programmes for enterprises. In particular, each ministry and local government provide information separately to prevent asymmetry of information, and integrated sites are organised to enable immediate verification and support.

**Figure 10:**
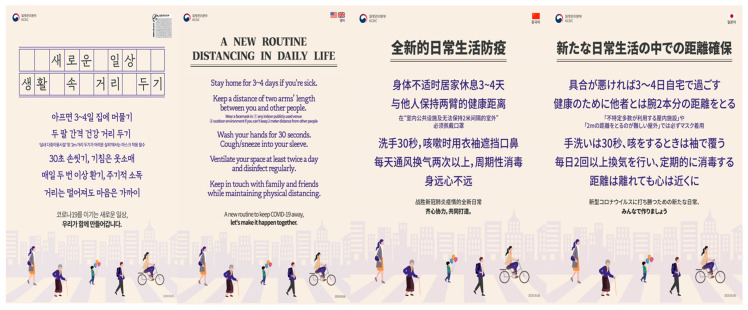
Routine Distancing Guideline.

In addition, emergency disaster support funds were established and disbursed throughout the nation. An amount of one million won for a four-person household was provided to stabilise people’s lives and restore the sagging economy. The government also provided subsidies for COVID-19 special unpaid leave. In the case of temporary leave of absence from a job that cannot be performed because of COVID-19, a “fast grant” is provided. The unpaid leave prompt support programme is a project that pays 500,000 won a month to workers whose income plummeted because of the pandemic—480 billion won was paid to about 320,000 people for up to three months. In addition, low-income families receive labor incentives—aimed at encouraging them to work and to supplement their household income—and children’s incentives—which are paid to help them raise their children. To this effect, workers were paid up to three million won, while families received 700,000 won per child. In addition, the government implemented a one-year grace period for the repayment of principal to individual debtors who faced difficulty repaying loans because of COVID-19 damages and provided management stabilisation funds to small business owners at low interest rates through small business rental support projects. Local government also launched various support programmes. For instance, the Seoul Metropolitan Government said it would pay 700,000 won to small business owners in a total of 1.4 million won over a two-month period. In particular, the Korean government continued to warn and guide, repeatedly emphasising that the transition to “routine distancing” was not a message to revert to a pre-COVID-19 lifestyle, but to establish new social norms with incorporated social distancing in daily life (See Figure 10). It continued to remind citizens to wear masks and keep themselves safe in order to contain the pandemic. These messages give the public a common goal—as in the time of war—and polls show that the majority of people support the government’s efforts. They did not succumb to panic or resort to hoarding because they trust the government of the day. This public trust is the driving force behind governmental efforts to promote civic awareness and voluntary cooperation.

## 5. Discussions

Korea’s COVID-19 disaster management was analysed in four stages and reconstructed into a single mechanism. In particular, the characteristics existing at each stage were analysed and the link between stages was assessed by considering the characteristics of each stage [11,12]. The study used a rough model based on the classification of factors affecting the disaster response presented to carry out a meta-analysis of COVID-19 policy cases [13]. In summary, after establishing the initial model, several rounds of meta-analysis were performed to refine it. Thus, the incomplete preliminary theoretical framework, which can be seen as an early model, indicated the simplification and classification of key factors through existing disaster response research. The study aimed to present a refined model by performing continuous analysis based on an incomplete framework and modifying the factors and their relationships. A number of studies and cases have shown that the responsibilities assigned to various organisations and agencies [9,10,11,12,13,14,15], and the factors that actually affect disaster response, are regular. Therefore, the goal was to classify these elements according to timeline in terms of system theory based on these regular factors. The research model was used to analyse the Korean government’s disaster management system and policies.

The Korean government announced several policies to improve its infectious disease management system in response to COVID-19. Based on the government’s response policy, this study concluded that: first, the national control tower should develop a step-by-step strategy and improve methods of risk assessment and determination of the crisis stage by considering various factors so as to reduce the gap between the infectious disease crisis alert level and the actual response. To cope with a new infectious disease, updated manuals were needed based on the latest knowledge, and this study realised the need for these and for the equivalent control tower to manage the implementation of the process and allow for fluid decision-making to enable rapid analysis and proactive response to amend the crisis situation. To begin with, it was necessary to establish a mechanism for a collaborative work network with the central government’s national control tower and local governance. In particular, COVID-19, which overshadows the graveness of MERS or SARS, has led to the awareness that not only medical professionals but the general public too are alert to the threat of infectious diseases. In particular, it can be confirmed that collaboration between enterprises, local government, the scientific community and the KCDC is instrumental in business consultations and partnerships between local government and the KCDC, which serve to provide the national control tower with resources such as mask production, testing kits and test result processing, and communication technology such as mobile apps like ‘CoronaMap’ to inform the public of tracking. Quintessential is the prevention of further spread, more important than anything else in managing infectious diseases.

While any one component of the model is difficult to isolate and verify in terms of impact and containment effectiveness, comparison to other countries and specific policies can provide a proxy. In terms of the national control tower model, Singapore is similar to Korea in terms of its outbreak, preparation and development of a national pandemic plan based on risk assessment and calibrated response measures. Singapore established the National Centre for Infectious diseases with a Multi-Ministry Task Force set up prior to COVID-19 to provide central co-ordination by the whole government [31,32]. Singapore likewise also scaled up rapid testing to 2200 a day for a population of 5.7 million—A similar ratio to Korea, with all suspected cases isolated, extensive contact tracing and quarantine with surveillance. Singapore’s containment effort was dubbed by the WHO as an “all government approach”. On the opposite side of the spectrum, Switzerland, which is also known as a small state with strong governmental control, has emphasized the value of testing, contract tracing and isolation as of March, 2020 [33,34,35].

Iceland can be a unique proxy for the effectiveness of early detection and wide-spread testing. Similar to other developed nations, Iceland initially established an eligibility-criteria through the health care system, with testing restricted to the highest risk populations either demonstrating symptoms or with confirmed contact with COVID-19 [36,37,38]. Taiwan has a similar stringently controlled border control with extensive efforts to contain the importation of disease via travel advisories, flight bans, entry restrictions, airport screening equipped with infrared fever screening, and home quarantine managed by the Taiwan Centre for Disease Control. Similarly, any travellers from abroad are isolated with thorough contact tracing within 24 h and home isolation for 14 days. Local health authorities check twice daily with symptomatic individuals sent to hospital. Stringent border controls have led to transparency in the importation of cases and is one policy that has significantly helped abate community transmission [39]. Taking isolation and quarantine one step further, New Zealand, which recently had a second wave despite stringent border controls, announced that after 102 days straight of no new cases the second wave has prompted a new set of more stringent isolation and quarantine measures. As of 13 August 2020, 32 hotels have been set up as facilities to isolate and quarantine all COVID-19 infected citizens and some relatives will be taken to mandatory quarantine with ‘tourism indefinitely cancelled’ [40,41].

In other words, countries that successfully manage COVID-19 are leading public-private partnerships through national control. Therefore, specific and systematic national disaster management is paramount to government. Through the case study of Korea, we seek to link theory with practice and to present a refined model by associating theoretical actions with real policies and factors. While democratic capitalist countries have discounted the importance of the role of government intervention, we maintain the need for a coordinated disaster response plan that can only be performed with effective and efficient mechanisms enabled by an empowered national government. Therefore, our research objective was to classify events so as to reconstruct and present the details in a manner and according to a timeline that links disaster management theory to actual government response. The research model utilised the Korean government’s disaster management system and policies to demonstrate the effective and irreplaceable role of the government. This study also contributes to the ideology that governments can be effective mechanisms and forces for good and that such a role can be aligned with democratic capitalist ideals respecting free media and serve the greater public good in times of crisis.

Therefore, the need for establishing an “infectious response network” involving the national control tower and the KCDC, local government and medical institutions was identified. Furthermore, the national disaster management system should establish epidemiological investigation methodologies after the occurrence of a disaster and systematically construct epidemiological surveys. COVID-19 is not controlled by genetic technology or advanced medicines, but by traditional measures such as epidemiological investigations, isolation, and quarantine. Therefore, it was recognised that the management of differentiated quarantine measures was important at each step of disaster response. Finally, the government should pay attention to preventing infectious diseases in everyday life, including overseas trips, and practice preventive measures, such as the National Action Guidelines, based on mature civic awareness. In addition, cooperation should be requested for investigation and preventive measures necessary to prevent the spread of infectious diseases. This study is expected to be path-breaking in this field as there are not many related studies in the case of the current pandemic, despite COVID-19-inspired awareness of the need to systematically manage disasters at a national level. In particular, the case study of the Korean government’s national disaster management analysed the role of the government from the initial phase to the active phase of response to disaster. Future post-response quantitative studies based on case studies and research models in other countries are expected to be relevant.

## 6. Conclusions and Implications

**Figure 11:**
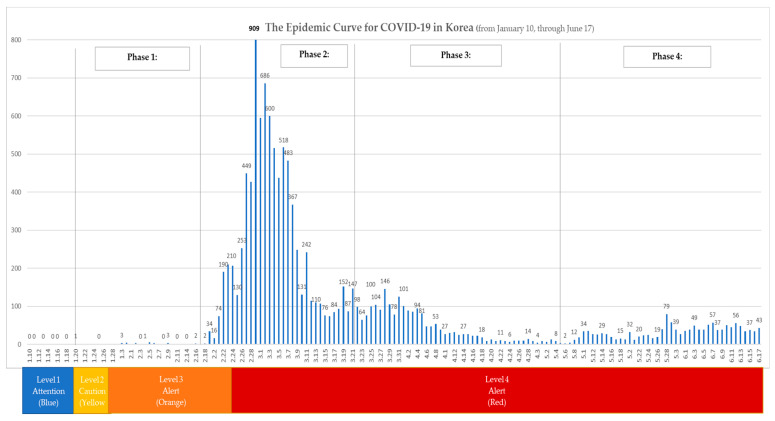
The Epidemic Curve for COVID-19 in Korea.

The purpose of this study is present the COVID-19 crisis in terms of the disaster management lifecycle and to specifically look at a national perspective to judiciously explore the factors that have enabled Korea. from identification of the first case through cluster and community transmission to what we term as near sustainable containment of COVID-19. In contrast to much traditional literature and the fundamental guiding values of western capitalism that purport a limited view of government, we argue that, similar to a wartime undertaking, pandemics such as COVID-19 require coordination and response that can only be carried out at a national level. While disaster management is widely studied, there is a void in studies analyzing the role of government in managing national disasters in general and of specific practices that have led to sustainable containment. While we recognize that there are a number of potential models to attain a level of containment, few studies have evaluated a long-term sustainable approach with specifics that enable the functioning of society on an economic level that is sustainable and avoids lockdown. Evidenced in Figure 11, Korea’s disaster management system based on control tower mechanism has led to a system that enables containment and that is somewhat sustainable as daily life with social distancing is allowed to proceed. While the current level does not indicate containment, clear testing and identification of cases, tracking and quarantine coupled with a health care treatment protocol provides some semblance of order and reassurance.

This study reconstructed a real lifecycle model based on the factors affecting the disaster response presented and is based on a meta-analysis of COVID-19 policy cases. To this end, through data specially requested from the Korean National Assembly, we applied a daily timeline model to traditional disaster management models by dividing events into four phases that are presented in a single timeline. The characteristics existing at each stage were analysed along with the indications for specific demarcated stages considering specific events that signified a major change in policies. Application of a traditional model with demarcated time periods and detailed actions of the Korean Government provides insight into how Korea, a country which identified its first COVID-19 case on January 20th, managed the situation Korea as noted has been praised by the WHO and is currently working with the UN on sharing of best practice on COVID-19 management.

The Korean government announced several policies to improve its infectious disease management system in response to COVID-19. Based on the government’s response policy, this study concluded that: first, disaster management should develop a step-by-step strategy and improve methods of risk assessment and determination of crisis stage by considering various factors, to reduce the gap between the infectious disease crisis alert level and the actual response. To cope with a new infectious disease, such steps include review and implementation of emergency response manuals and the need for nationally coordinated leadership and establishment of a mechanism that coordinates various functions akin to the central government’s national control tower alongside local governance. In particular, COVID-19, which overshadows the graveness of MERS and SARS, increased the need for a central coordinating effort and, we believe, consideration of the role of the national government as a central coordinating force.

Second, in disaster management upon establishment of command and control, one of the primary objectives of the initial response is the amelioration of public fear and the setting of a tone and approach to leadership. This includes communication that is aligned with public awareness and public health. At this stage, ideally leadership implements initial emergency relief plans and formulates a coordinated communication strategy. To the end, by 5 February, the Korean Government had launched a website and initiated daily press briefings – to date, posting over 176 daily briefings and 96 other briefings and documents for public viewing. Under the command and control system, the Korea Center for Disease Control at the helm of decision-making from the Control Tower. The KCDC also decided on a full non-disclosure of information which includes the engagement of industry and empowerment stakeholders including doctors, researchers, the scientific community and industry, with a firm commitment to base responses on fact and medical knowledge rather than politics. A second example is the establishment of the mask stabilisation committee which was convened and was key to the production, supply, price control and enforcement of mask usage in public, a cornerstone effort that enabled Korea to continue to use mass transit such as the subway system and for businesses to operate with social distancing. These actions demonstrate leadership and the ability to quickly identify specific action areas critical to the prevention of mass contagion.

The third step is leadership to identify, engage and empower stakeholders. In times of crisis, engagement of stakeholders such as private IT developers, healthcare workers, private enterprises, and the scientific community assures that resources are put in place to best identify and respond in an effective and efficient manner, for example, the effort to empower private enterprise to develop COVID-19 test kits and testing solutions. Such efforts resulted in a two-fold positive effective. First, as seen from the timeline, the collaborative approach enabled multiple COVID-19 tests to be developed along with development of innovations such as drive-thru testing, to reduce the need for sanitisation measures, and of walk through testing sites with negative pressure booths. As a result, Korea is a leader in exporting test kits to more than 100 countries, leading to over 120 million dollars in export revenue per month.

Fourth, we examined a particular COVID containment strategy. In the case of Korea, the national disaster management system enacted a test, trace and quarantine system. Early identification of COVID-19 positive individuals, many of whom may be asymptomatic and not requiring treatment, is critical. Korea also utilised the ability to legally conduct epidemiological investigation to systematically track the movement of COVID-19 positive patients. While we recognise this is a controversial point, tracing and tracking introduces an issue of public good versus private benefit and a trade-off between these. By enabling the government to track and utilise existing technology such as GPS data and surveillance, there is a degree of accuracy and procedures for every COVID-19 positive case. This effort and these actions, which complement the quarantine and interview process and the quick turnaround of testing, enable accurate reconstruction of epidemiological paths. Therefore, we recognised that the management and implementation of measures to test, trace and quarantine are quintessential elements in Korea’s disaster response and ability to avoid lockdown.

Fifth is effective implementation. Through in-depth interviews and focus groups, we confirmed that Korea has had exceptional follow through including border control which involves isolation and quarantine of entire flights in off-site locations that are segmented by age with older passengers going to more comfortable facilities than younger passengers, private transportation from the airport to testing centers or to homes for quarantine with instructions, and two daily calls to every individual in quarantine, which is in addition to mandatory login to a mobile app and GPS tracking overseen with a local case officer. Additionally, the authors have witnessed posters in their residences with pictures indicating the sterilisation of the building after a COVID+ patient was identified, the closing of retail locations upon epidemiological tracking, hand sanitizer in virtually every elevator and grocery store, and temperature checks at most public and private locations upon entry. Such stringent implementation ensures compliance and restores faith in the measures, policies and actions the government takes on behalf of society. Combined with social distancing, nearly 20 daily text messages, numerous apps to trace COVID patients, mask supply, and KCDC daily updates, the public is kept informed and the Korean Government has provided sufficient information and follow through measures to ensure effective implementation and to cultivate mature civic awareness. This latter is quintessential as ultimately compliance and widespread buy-in is essential as collective awareness and effort is necessary to prevent the spread of infectious diseases.

This study is expected to be path-breaking in this field as there are not many related studies in the case of the current pandemic, despite COVID-19-inspired awareness of the need for systematic management of disasters at a national level. In particular, the case study of the Korean government’s national disaster management analyzed the role of the government from mitigation and planning phases to execution, with details of specific policies and actions in response phases to the beginning of what we term the sustainable containment phase of disaster management. Future post-response quantitative studies based on case studies and research models in other countries are expected to be useful. Akin to the role of national defence, in disaster cases the government has a critical role in containment and stabilisation of the country. Lastly, in terms of disaster management, should this be effectively managed the last phase of containment and recovery is attainable. This study presents the successful national disaster management system of the Korean government. However, considering the nature of the COVID-19 virus, there is still a possibility of the pandemic reoccurring; thus, sustainable management is required until the virus is conquered.

## Figures and Tables

**Table 1:** Cases and Testing in Korea as of 29 July 2020, data aggregated from January 20.

Korean Population	Tests Conducted with Results	Confirmed Cases	Currently in Isolation or Quarantine	Released from Isolation / Quarantine	Deceased
51,845,612	1,547,307	14,251	882	13,069	300
Data as of 29 July 2020	2.89% Total Population	0.9% Positivity Rate			2.11% Deceased / positivity
**Regional Breakdown of Confirmed Cases** 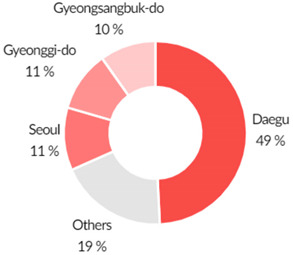			**Breakdown of Cluster Outbreaks** 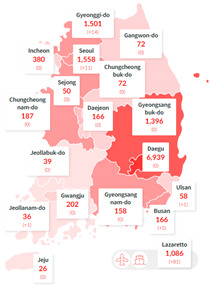		

Source: Central Decease Control Headquarters, Republic of Korea.

## Supplementary Materials

The following are available online at https://www.mdpi.com/1660-4601/17/18/6691/s1, Table S1: Korea Crisis Management Timeline, Table S2: Korea Crisis Management phase events.
