# Supplementary material for: National Disaster Management System: COVID-19 Case in Korea
Source: Int J Environ Res Public Health. 2020 Sep 14;17(18):6691. doi: 10.3390/ijerph17186691 (PMC7559530; doi:10.3390/ijerph17186691)
Supplement: Supplementary file 1 [file ijerph-17-06691-s001.pdf]

Supplementary Data

Table 1. Korea Crisis Management Timeline.

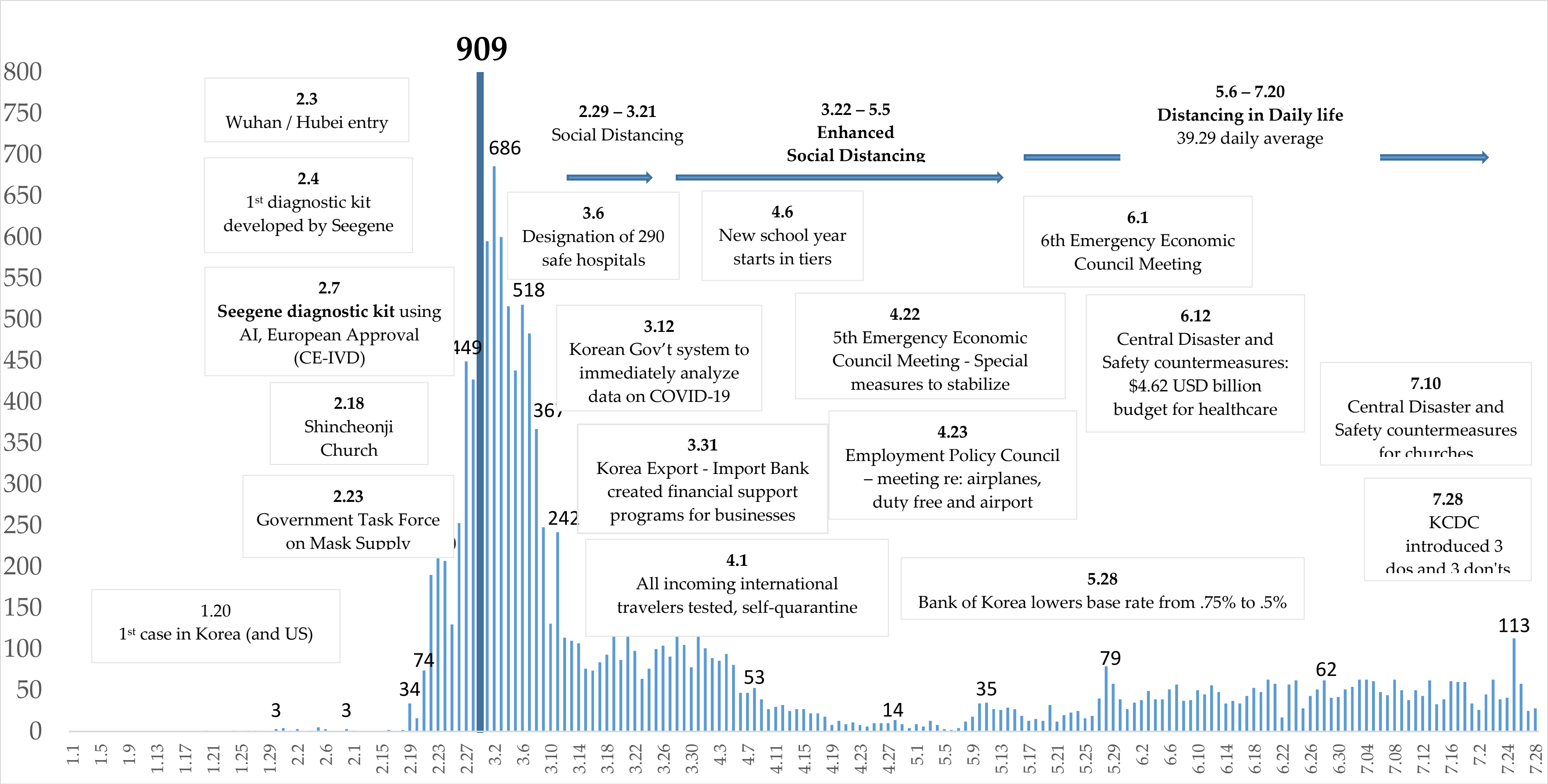

Before outbreak ~ 19 Jan	1st Phase: 1st case to outbreak 20 Jan – 18 Feb	2nd Phase: community transmission 19 Feb – 21 March	3rd Phase: Social Distancing 22 March – 5 May	4th Phase: Routine Distancing 6 May - current

Table S2. Korea Crisis Management phase events

	1st Phase		2nd Phase		3rd Phase		4th Phase	
Dates	20 January – 18 February		19 February – 21 March		22 March – 5 May		6 May - Current	
Characteristics of outbreaks	Imported cases		Large-scale clusters social distancing period		Enhanced social distancing		Distancing in daily life local clusters, sporadic cases	
Major Responses	1.27	Crisis Alert raised level 2 → level 3	2.23	Crisis Alert raised level 3 → level 4	3.22 ~	Enhanced Social Distancing	5.6 ~	Distancing in daily life
	2.4	Special entry screen from Hubei / Wuhan	2.26	Ministry of Economy and Finance convened a Task Force of Mask Supply Stabilisation measures	3.31	Korea Export – Import bank offered financial support for business	5.28	Bank of Korea lowered base rate from .75% to .5%
	2.7	Seegene develops virus diagnostic with AI, receives European approval (CE-IVD)	3.1	Focus on treatment of severe cases. Establishment of life treatment centers for mild cases	4.1 ~	Strengthened management of incoming international travelers	5.29	Strengthening measures for Seoul metropolitan daily area
	2.11	Co100 app launched using government data and alerts	3.4 3.7	Korean Government developed self-quarantine app for Android and iOS	4.1	Government collaborated with Korean Telecom (KT) to make foot traffic and roaming data available	6.2	Designated high risk countries that require strengthening infectious disease control and prevention
			3.11	Private developers launched appl for mask retailers	4.22	5th Emergency Economic Council meeting established measures and allocated $10 billion USD	6.12	Central Disaster Safety & Countermeasure HQ meeting: $4.62 billion USD budget for healthcare infection
			3.1	Focus on treatment of severe cases and establishment of life treatment centers for mild cases			7.10	Central Disaster Safety & Countermeasure announced mandatory prevention guidelines for churches
Major cases and clusters and accomplishments	• Imported cases (17)<br>• Secondary transmission (13)		• Daegu Shincheonji Church (5,213)<br>• Self-quarantine app developed by 4.13, 91.4% quarantined installed.<br>• Designation of 290 safe hospitals<br>• Identification & testing of 9,334 Shincheonji Church members in Daegu		• Cheongdo Daenam Hospital (121)<br>• Guro-Gu call center (169)<br>• By 3.30 tested 250,000 at 600 testing sites, capacity to test 20,000 / day with results within 6 hours via text messages.		• Nightlife clubs Itaewon Gay Night Club (277)<br>• Door-to Door sales – Korea (210)<br>• Religious facilities Seoul Metropolitan churches (119)<br>• 7.20 last two weeks 59.5% cases imported with local clusters 24.4%	
